# Raloxifene inhibits the overexpression of TGF-β1 in cartilage and regulates the metabolism of subchondral bone in rats with osteoporotic osteoarthritis

**DOI:** 10.17305/bjbms.2020.5142

**Published:** 2021-06

**Authors:** Shao-Hua Ping, Fa-Ming Tian, Hao Liu, Qi Sun, Li-Tao Shao, Qiang-Qiang Lian, Liu Zhang

**Affiliations:** 1 Department of Orthopedic Surgery, Hebei Medical University, Shijiazhuang, China; 2 Medical Research Center, North China University of Science and Technology, Tangshan, China; 3 Department of Orthopedic Surgery, Affiliated Hospital of North China University of Science and Technology, Tangshan, China; 4 Department of Orthopedic Surgery, Emergency General Hospital, Beijing, China

**Keywords:** Osteoporosis, osteoarthritis, raloxifene, TGF-β1, cartilage, subchondral bone

## Abstract

Overexpression of transforming growth factor-beta 1 (TGF-β1) and subchondral bone remodeling play key roles in osteoarthritis (OA). Raloxifene (RAL) reduces the serum level of TGF-β1 in postmenopausal women. However, the effect of RAL on TGF-β1 expression in articular cartilage remains unclear. Therefore, we aimed to investigate the protective effect of RAL against osteoporotic OA mediated by TGF-β1 expression in the cartilage and the metabolism of subchondral bone. Osteoporotic OA was induced by a combination of anterior cruciate ligament transection (ACLT) and ovariectomy (OVX). Rats were divided into five groups (n = 12): the sham, ACLT, OVX, ACLT + OVX, and RAL groups (ACLT + OVX + RAL, 6.25 mg/kg/day for 12 weeks). Assessment was performed by histomorphology, microcomputed tomography (micro-CT), immunohistochemistry, and tartrate-resistant acid phosphatase (TRAP) staining. Extreme cartilage degeneration was detected in the ACLT + OVX group. The histomorphological scores, levels of TGF-β1, and its related catabolic enzymes and osteoclasts numbers in the ACLT + OVX group were higher than those in other groups (*p* < 0.05). Furthermore, the structure model index (SMI) and trabecular spacing (Tb.Sp) were decreased (*p* < 0.05), while the bone mineral density (BMD), bone volume fraction (BV/TV), and trabecular number (Tb.N) were increased after treatment with RAL compared with the corresponding parameters in the ACLT + OVX group (*p* < 0.05). Our findings demonstrated that RAL at clinical doses retards the development of osteoporotic OA associated with the inhibition of TGF-β1 overexpression in the cartilage and regulation of subchondral bone metabolism. These results suggest an expansion of the clinical indications for RAL to include the prevention and treatment of postmenopausal OA.

## INTRODUCTION

Osteoarthritis (OA) is the most common type of arthritis leading to joint disability in the elderly population and particularly in women worldwide [1]. Multifactorial etiologies and pathogenesis, including aging, overweight, hormone imbalance, and other factors, are involved in the progression of OA [2,3]. Osteoporosis (OP) is another prevalent musculoskeletal disorder characterized by bone loss and deteriorated microstructure of the bone tissue and is closely related to high incidence of OA in postmenopausal women [4,5]. The serum level of the specific degradation fragment of collagen type II (CTX-II) was shown to be increased in postmenopausal women indicating that estrogen deficiency has a catabolic effect on the cartilage [5]. Castañeda et al. found that estrogen deficiency may lead to OA via a decrease in the bone mass and a direct negative effect on the cartilage in ovariectomized rabbits [6]. OP aggravates cartilage degeneration by increasing the subchondral bone resorption in a model of OA [7]. These findings indicate that close associations of OA and OP are related to estrogen deficiency, which has a catabolic effect on the cartilage and subchondral bone. In recent years, estrogen and its replacement agents have been shown to play a protective role in OA via modulation of the subchondral bone turnover and direct beneficial effects on the cartilage [5].

Selective estrogen receptor modulators (SERMs) are estrogen replacement agents for postmenopausal OP that have dual protective effects on the cartilage and subchondral bone in OA [8]. Raloxifene (RAL) is the first SERM approved by the FDA for the treatment and prevention of OP [9]. The protective effect of RAL on chondrocytes has been recently demonstrated. Kavas found that RAL increased the expression levels of the aggrecan (*AGG*) and collagen type II (*Col-II*) genes and inhibited chondrocyte apoptosis by decreasing the expression of matrix metalloproteinases-13 (MMP-13) in OA chondrocytes [10]. In a clinical study, the serum level of CTX-II, a marker of cartilage degradation, was reduced in postmenopausal women treated with RAL [9]. These findings demonstrated that RAL has a potential therapeutic effect in OA. Based on these positive results, further studies on the therapeutic mechanism of RAL will provide support for its clinical application in the treatment of postmenopausal OA.

The transforming growth factor-beta (TGF-β) pathway plays a critical role in the development of OA due to the diphasic regulation of the cartilage metabolism [11-13]. TGF-β1, a member of the TGF-β superfamily, is an important regulatory cytokine that maintains the homeostasis of articular cartilage [14,15] by promoting chondrogenesis and production of cartilage extracellular matrix (ECM) [16,17]. However, it is worth noting that there are two TGF-β1 receptors, including the activin receptor-like kinases 5 (ALK5) and ALK1. TGF-β1 plays the anabolic and catabolic roles via the canonical ALK5/Smad2/3 pathway and noncanonical ALK1/Smad1/5/8 pathway in the cartilage [11,13]. The overexpression of TGF-β1 in the articular cartilage was detected in human OA samples [18] and in a rat model of osteoporotic OA [19] and is considered responsible for cartilage degeneration [20-22]. The negative effects of TGF-β1 may be related to a switch from the anabolic ALK5-Smad2/3 pathway to the catabolic ALK1-Smad1/5/8 pathway in OA cartilage [13]. Overall, these findings suggest that suppression of the overexpression of TGF-β1 may play a potential therapeutic role in OA. RAL was demonstrated to decrease the TGF-β1 level in experimental breast cancer cells [23] and reduce the serum level of TGF-β1 in postmenopausal women [24]. However, the regulatory effect of RAL on TGF-β1 expression in the cartilage in osteoporotic OA remains unclear.

In addition to cartilage degeneration, alteration of the subchondral bone is another important factor in the development of OA. Estrogen deficiency, an important risk factor for OA in early postmenopausal women, has negative effects on the subchondral bone mediated by high bone turnover [25]. Bone resorption inhibitors, including SERMs, were shown to have beneficial effects by improving the health of the subchondral bone [9].

This study was designed to evaluate the chondroprotective effect of RAL associated with TGF-β1 expression in the articular cartilage and alterations of the subchondral bone in a model of osteoporotic OA. The results support a potential expansion of the clinical indications of RAL to include postmenopausal OA.

## MATERIALS AND METHODS

### Study design

Sixty twelve-week-old female Sprague-Dawley rats (mean weight 268 g) (Changsheng Biotechnology Co., Ltd., Liaoning, China) were used in this study. OA and OP were induced by anterior cruciate ligament transection (ACLT) and ovariectomy (OVX), respectively. Before the operation, the animals were randomly divided into five groups of 12 rats each as follows: The sham group, ACLT group, OVX group, ACLT + OVX group, and RAL group (ACLT + OVX + RAL). Then, each group was randomly divided into two subgroups; one subgroup was used for macroscopic scoring and microcomputed tomography (micro-CT) analysis (n = 6) and another group was used for microscopic scoring and immunohistochemical evaluation (n = 6).

### Surgical procedures

Bilateral ovaries were exposed through dual dorsal incisions (2 cm long) and resected in the OVX group [26], and ACLT was performed via a medial incision on the right knee in the ACLT group [27]. Briefly, after lateral dislocation of the patella, ACL was identified and transected under visual inspection. An anterior drawer test was performed intraoperatively to confirm the completeness of the transection [27]. All incisions were closed after irrigation by saline. The RAL group and ACLT + OVX group received both OVX and ACLT, and the sham group underwent only sham operation.

### Drug treatment

RAL (Hengrui Medicine Co., Ltd., Jiangsu, China) was administered to the animals of the RAL group by oral gavage (6.25 mg/kg/day) daily at 72 hours after the surgery for 12 weeks. Other groups received the same volume of distilled water as a vehicle.

### Macroscopic and pathological scoring

After sacrifice, the right knees were disarticulated and imaged for macroscopic scoring. Then, the specimens were fixed in 100% ethanol for micro-CT analysis. Gross lesions of the cartilage in the tibial plateau were graded as described previously (Supplementary Table 1) [3].

The samples for pathological evaluation were harvested and instantly fixed in 10% neutral formalin for 72 hours. Then, decalcification was performed in 10% ethylenediaminetetraacetic acid (EDTA) for approximately 2 months. The decalcified specimens were embedded in paraffin and sagittal sections (6 μm thickness) were sequentially obtained from the weight-bearing zone of the medial compartment of the knee. Three nonsequential sections from each knee were stained with toluidine blue. Pathological evaluation was performed blindly based on the scoring system of the Osteoarthritis Research Society International (OARSI) by two independent observers experienced in pathological evaluation [28].

### Immunohistochemical evaluation

Immunohistochemical assessment was performed according to the procedures described previously [29]. Target proteins in the cartilage were detected using the following primary antibodies: TGF-β1(1:200)(ab92486, Abcam, Inc., USA), ADAMTS-5 (1:500)(BA3020, Boster Co., Ltd., China), Col-II (1:500) (P02458, Boster), MMP-13 (1:500)(P23097, Boster), collagen type X (Col-X,1:200)(bs-0554R-FITC, Bioss Co., Ltd., China), and AGG (1:500)(GTX54920, Gene Tex, Inc., USA). In addition, collagen type I (Col-I,1: 200) (BA0325, Boster) was assayed to evaluate the level of bone matrix in the subchondral bone. The assessment of the staining was based on the average sum of the integrated optical density (IOD/mm^2^), which is the ratio of the total IOD and the area of the region of interest (ROI) (mm^2^) [29]. The cartilage of the load-bearing zone was defined as ROI [30]. Measurements were performed using Image-Pro Plus version 6.0.0.260 (Media Cybernetics, Inc., USA).

### Micro-CT scan

All samples were analyzed by a Skyscan1176 micro-CT system (Bruker, Kontich, Belgium) with the voxel size of 18 mm. The trabecular region of the medial tibial plateau compartment was defined as a ROI [31]. The following parameters were used to evaluate the bone mass and the microstructure of the subchondral bone: Bone mineral density (BMD, mg/cm^3^), trabecular bone volume (BV/TV, %), trabecular number (Tb.N, 1/mm), trabecular thickness (Tb.Th, mm), structure model index (SMI), and trabecular separation (Tb.Sp, mm). All parameters were calculated by CT Analyzer version 1.14.4.1 (Skyscan, Kontich, Belgium).

### Tartrate-resistant acid phosphatase (TRAP) staining

TRAP staining was performed to evaluate the osteoclast activity according to the standard instructions of the manufacturer (Lianke Biotech, Hangzhou, China). The number of osteoclasts in the subchondral bone of each sample was counted as described previously [32].

### Ethical statement

All experimental protocols were approved by the Laboratory Animal Ethical Committee of North China University of Science and Technology (approval number: LX2018156).

### Statistical analysis

Data of IOD, micro-CT, and TRAP data are presented as the mean ± standard deviation and were evaluated using one-way analysis of variance (ANOVA), followed by Fisher’s least significant difference (LSD) t-test or Dunnett’s T3 test. Scores are reported as the median (quartiles) and were analyzed by nonparametric tests (Kruskal–Wallis and Mann–Whitney). *p* < 0.05 was used to indicate statistical significance. All data were analyzed with SPSS 22.0 (IBM Corp., Armonk, NY, USA).

## RESULTS

### RAL retarded cartilage degeneration in osteoporotic OA

**FIGURE 1:**
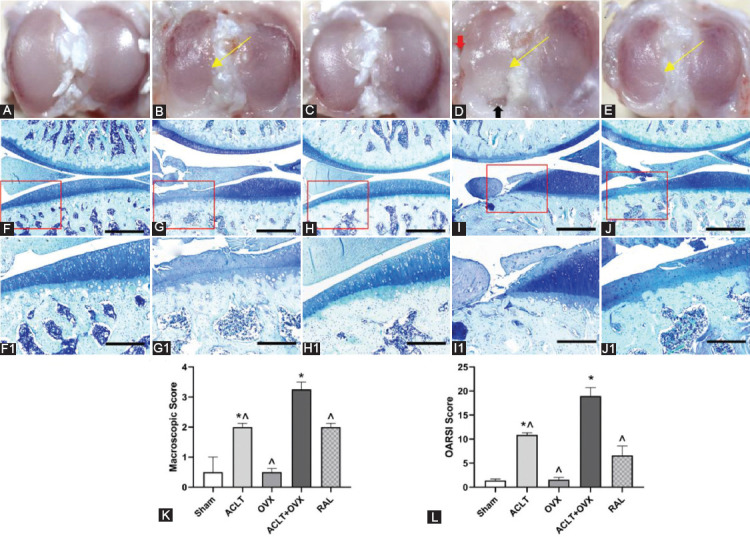
RAL retarded cartilage degeneration in osteoporotic OA. Macroscopic views of articular cartilage: (A) The sham group, smooth cartilage surface; (B) The ACLT group, erosions in the cartilage (yellow arrow); (C) The OVX group, essentially smooth surface; (D) The ACLT + OVX group, serious lesions (yellow arrow) with a zone of complete wear of the cartilage (black arrow) and cartilage proliferation (red arrow); (E) The RAL group, alleviated erosion versus the ACLT + OVX group (yellow arrow). Images of toluidine blue staining (scale bar, 500 μm): (F) The sham group, normal cartilage; (G) The ACLT group, lesions almost reaching the deep layer of the cartilage; (H) The OVX group, essentially normal cartilage; (I) The ACLT + OVX group, serious lesions with exposure of the subchondral bone; (J) The RAL group, lesions reaching the middle layer of the cartilage. (F1) - (J1): The magnified images of the cartilage erosion zone in the corresponding boxes in (F) - (J) (scale bar, 200 μm). (K) The macroscopic scores of all groups. (L) The OARSI scores. ^*p* < 0.05 versus the ACLT + OVX group;*p < 0.05 versus the sham group. ACLT: Anterior cruciate ligament transection; OVX: Ovariectomy; RAL: Raloxifene; OARSI: Osteoarthritis Research Society International.

The articular surface in the sham (Figure 1A) and OVX groups (Figure 1C) was smooth; however, lesions in the cartilage of the medial tibial plateau were observed in the ACLT group (Figure 1B). Deteriorated lesions, cartilage proliferation, and even subchondral bone exposure were detected in the ACLT + OVX group (Figure 1D). Cartilage degeneration was alleviated by RAL (Figure 1E) compared with that in the ACLT + OVX group. The macroscopic scores are illustrated in Figure 1K. The score in the ACLT group was higher than that in the sham group (*p* < 0.005) but lower than that in the ACLT + OVX group (*p* < 0.005). The score in the RAL group was significantly lower than that in the ACLT + OVX group (*p* < 0.005). Microscopic assessment indicated that articular cartilage in the sham group (Figure 1F and F1) is normal. Mild wrinkles or superficial fissures in the surface of cartilage surface were observed in the OVX group (Figure 1H and H1). In the ACLT (Figure 1G and G1) and the RAL groups (Figure 1J and J1), cartilage erosions into the middle layer were detected. However, extensive cartilage defects and subchondral bone exposure were present in the ACLT + OVX group (Figure 1I and I1). As shown in Figure 1L, the OARSI scores in the ACLT group were higher than that in the sham group (*p* < 0.005) but lower than that in the ACLT + OVX group (*p* < 0.005). RAL decreased the OARSI score compared with that in the ACLT + OVX group (*p* < 0.001). No significant differences in both scores between the OVX and sham groups were detected (*p* > 0.05).

### RAL regulated the levels of metabolic factors and preserved the matrix of cartilage and bone

**FIGURE 2:**
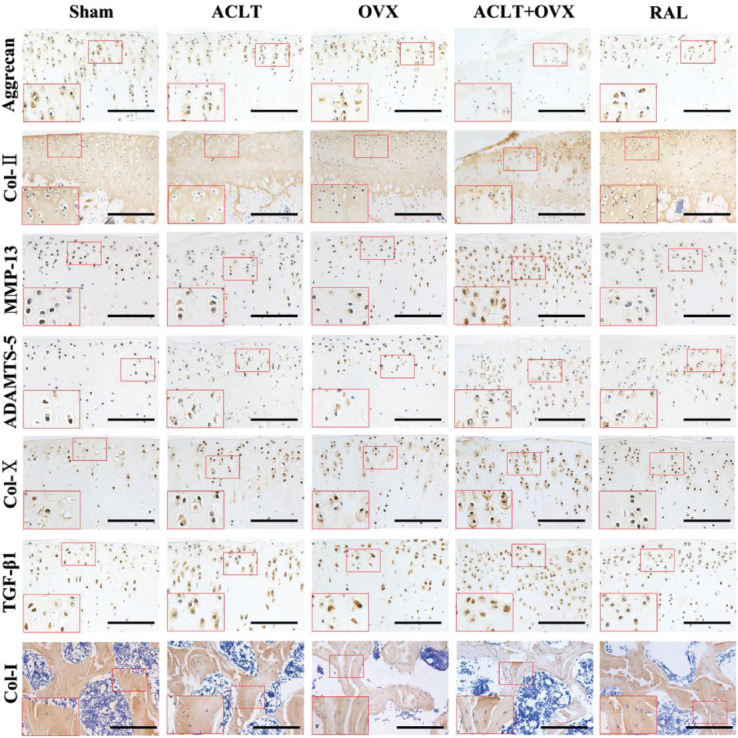
RAL preserved the matrix of cartilage and bone and inhibited the overexpression of TGF-β1 and catabolic factors. Expression of aggrecan, Col-II, MMP-13, ADAMTS-5, Col-X, TGF-β1, and Col-I (scale bar, 100 μm). Col-II: Collagen type II; MMP-13: Matrix metalloproteinase-13; ADAMTS-5: A disintegrin and metalloproteinase with thrombospondin motifs-5; Col-X: Collagen type X; TGF-β1: Transforming growth factor-beta 1; Col-I: Collagen type I.

**FIGURE 3:**
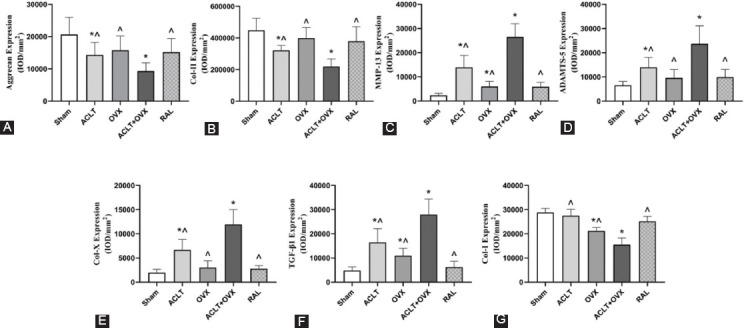
Statistical analysis of the results of immunohistochemistry. The quantified protein levels are listed as follows (IOD/mm^2^): (A) Aggrecan; (B) Col-II; (C) MMP-13; (D) ADAMTS-5; (E) Col-X; (F) TGF-β1; and (G) Col-I. ^*p* < 0.05 versus the ACLT + OVX group;*p < 0.05 versus the sham group. Col-II: Collagen type II; MMP-13: Matrix metalloproteinase-13; ADAMTS-5: A disintegrin and metalloproteinase with thrombospondin motifs-5; Col-X: Collagen type X; TGF-β1: Transforming growth factor-beta 1; Col-I: Collagen type I; IOD: Integrated optical density.

Immunohistochemical staining images are shown in Figure 2, and the results of average IOD are illustrated in Figure 3A-G. The levels of MMP-13, ADAMTS-5, COL-X, and TGF-β1 in the ACLT group were significantly higher than those in the sham group (all *p* < 0.05) but lower than those in the ACLT + OVX group (all *p* < 0.05). However, the levels of these factors were substantially decreased after RAL treatment compared with those in the ACLT + OVX group (all *p* < 0.05). The levels of Col-II (*p* < 0.005) and AGG (*p* < 0.05) in the ACLT group were lower than those in the sham group. Significantly decreased levels of Col-II (*p* < 0.05) and AGG (*p* < 0.05) were observed in the ACLT + OVX group compared with those in the ACLT group. As expected, RAL increased the expression of Col-II (*p* < 0.001) and AGG (*p* < 0.05) compared with those in the ACLT + OVX group. In addition, the level of Col-I in the OVX group was significantly lower than that in the sham group (*p* < 0.001) but considerably higher than that in the ACLT + OVX group (*p* < 0.001). Conversely, RAL increased the level of Col-I compared with that in the ACLT + OVX group (*p* < 0.001). No significant differences were detected between the ACLT and sham groups (*p* > 0.05).

### RAL inhibited bone loss and improved subchondral bone health

**FIGURE 4:**
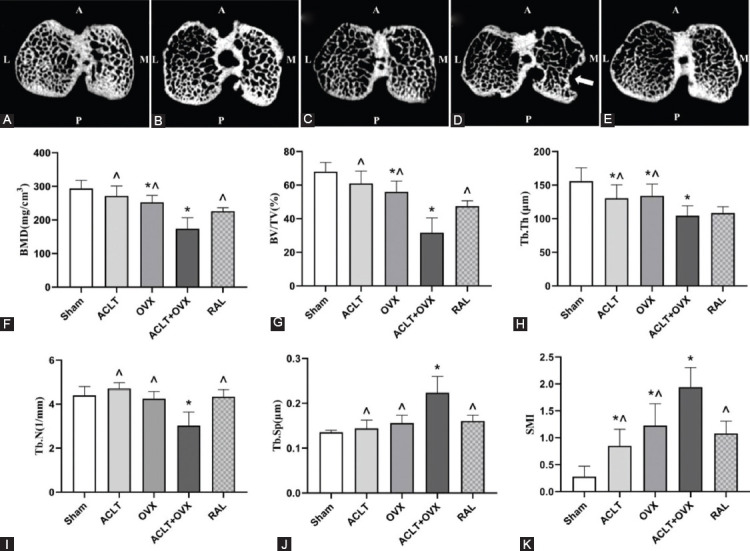
RAL improved the health of the subchondral bone. Transaxial images of the subchondral bone of micro-CT scans: (A) The sham group, normal trabecular; (B) The ACLT group, reduced trabecular thickness, and damaged microstructure; (C) The OVX group, bone resorption; (D) The ACLT + OVX group, considerable bone resorption with bone defect (white arrow); and (E) The RAL group, improved microstructure versus the ACLT + OVX group. The microstructural parameters are shown as follows: (F) BMD; (G) BV/TV; (H) Tb.Th; (I) Tb.N; (J) Tb.Sp; and (K) SMI. ^*p* < 0.05 versus the ACLT + OVX group; *p < 0.05 versus the sham group. ACLT: Anterior cruciate ligament transection; OVX: Ovariectomy; RAL: Raloxifene; BMD: Bone mineral density; BV/TV: Bone volume fraction; Tb.Th: Trabecular thickness; Tb.N: Trabecular number; Tb.Sp: Trabecular spacing; SMI: Structure model index.

Representative traverse images of the subchondral bone are shown in Figure 4A-E, and the results of various analyses are illustrated in Figure 4F-K. Lower Tb.Th (*p* < 0.05) and higher SMI (*p* < 0.005) were detected in the ACLT group compared with those in the sham group. BMD (*p* < 0.01), BV/TV (*p* < 0.005), and Tb.Th (*p* < 0.05) were significantly decreased and SMI (*p* < 0.001) was substantially increased in the OVX group compared with those in the sham group. However, BMD, BV/TV, Tb.Th, and Tb.N were strongly decreased, and SMI and Tb.Sp were increased in the ACLT + OVX group compared with those in the ACLT and the OVX groups, respectively (all *p* < 0.05). RAL significantly increased BMD (*p* < 0.01), BV/TV (*p* < 0.001), and Tb.N (*p* < 0.05) and decreased SMI (*p* < 0.001) and Tb.Sp (*p* < 0.05) compared with those in the ACLT + OVX group.

### RAL inhibited the activity of osteoclasts

**FIGURE 5:**
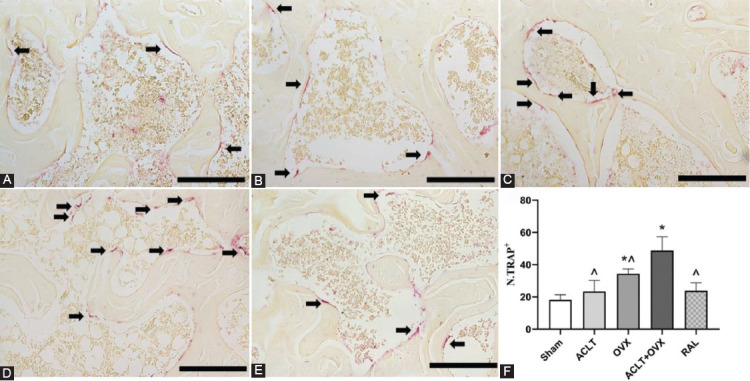
RAL inhibited the activity of osteoclasts in the subchondral bone. TRAP + osteoclasts are indicated by black arrows (scale bar, 100 μm): (A) The sham group; (B) The ACLT group; (C) The OVX group; (D) The ACLT + OVX group; and (E) The RAL group. (F) The number of TRAP-positive osteoclasts (N.TRAP+) in all groups. ^*p* < 0.05 versus the ACLT + OVX group; *p < 0.05 versus the sham group. ACLT: Anterior cruciate ligament transection; OVX: Ovariectomy; RAL: Raloxifene; TRAP: Tartrate-resistant acid phosphatase; N.TRAP+: The number of TRAP-positive osteoclasts.

As shown in Figure 5F, no differences in the number of osteoclasts were detected between the ACLT (Figure 5B) and sham groups (Figure 5A) (*p* > 0.05). The number of osteoclasts was higher in the OVX group (Figure 5C) than that in the sham group (*p* < 0.05) and lower than that in the ACLT + OVX group (Figure 5D) (*p* < 0.001). RAL (Figure 5E) substantially decreased the number of osteoclasts compared with that in the ACLT + OVX group (*p* < 0.001).

## DISCUSSION

The development of OA cannot be stopped [33]. However, bone metabolism regulators, including SERMs, delay the progression of OA [9,34]. In a previous study, a high dose of RAL (10 mg/kg/day) used in a patellofemoral joint model of OA raised concerns about an increase in the incidence of complications, as mentioned by the author [35]. Therefore, in this study, RAL was administered at a dosage equivalent to that in humans. Moreover, early administration was shown to induce better chondroprotective effects in previous studies [32,36]. Hence, in the present study, gavage was started early after the surgery. According to the macroscopic and pathological analysis in this study, OVX considerably aggravated the cartilage destruction induced by ACLT, which is in agreement with the results of previous studies [7,27]. The potential mechanism of this effect may be related to the overexpression of TGF-β1 in the cartilage and aggravation of the subchondral bone remodeling by estrogen deficiency. Conversely, alleviation of the cartilage destruction after RAL treatment may be associated with inhibition of overexpression of TGF-β1 in the cartilage and improvement of trabecular health. Therefore, our findings indicate that early administration of RAL retards the cartilage degeneration and improves the health of the subchondral bone in osteoporotic OA.

The homeostasis and integrity of the articular cartilage are maintained by the ECM, including Col-II and AGG [37]. MMP-13 and ADAMTS-5 are important catabolic enzymes that degrade the ECM during the progression of OA [37,38]. Destruction of the ECM results in terminal differentiation and hypertrophy of chondrocytes prior to apoptosis characterized by increased expression of Col-X [39]. TGF-β1 was demonstrated to protect chondrocytes, and a lack of TGF-β1 induces hypertrophy and terminal differentiation of chondrocytes [16,17,40]. However, the negative effects of TGF-β1, including induction of MMP-13, ADAMTS-5, and Col-X, were shown to induce cartilage degeneration [13,41]. In this study, substantially increased levels of TGF-β1, MMP-13, and Col-X were observed in the ACLT group, similar to the data of a previous study [42], and the levels of these cytokines and ADAMTS-5 were considerably higher in the ACLT + OVX group compared with those in the ACLT group. These findings indicate that overexpression of TGF-β1 is probably involved in the mechanism of aggravation of cartilage degeneration by OVX, which has not been reported previously. Mechanical stress on the cartilage is expected to trigger the activation of TGF-β1 [15]; hence, abnormal postoperative stress on the cartilage may be involved in the potential mechanism of TGF-β1 overexpression in the ACLT group. Furthermore, the microstructural damage of the subchondral bone decreases stress dispersion and strengthens abnormal shear stress on the cartilage [2], thus possibly enhancing the overexpression of TGF-β1. Therefore, deterioration of the damaged trabecular microstructure by estrogen deficiency may be responsible for the aggravation of abnormal stress on the cartilage, thus potentially increasing the level of TGF-β1 in the ACLT + OVX group. High concentrations of TGF-β1 can induce a shift from the ALK/Smad2/3 to ALK1/Smad1/5/8 pathway [13] to promote the production of MMP13 and ADAMTS-5, thus inducing cartilage degeneration [22]. Therefore, in this study, enhanced overexpression of TGF-β1 indirectly induced by estrogen deficiency and biphasic regulation of the ALK/Smad pathways by TGF-β1 may be involved, at least partly, in the potential mechanism of the aggravated cartilage degeneration in the model of osteoporotic OA used in this study. However, additional molecular and biomechanical studies are required to confirm this hypothesis.

In this study, RAL inhibited the overexpression of TGF-β1 and associated catabolic enzymes in the cartilage of osteoporotic OA. Chondroprotective effects mediated by the suppression of TGF-β1 expression have been reported in previous studies [41,42]. Thus, inhibition of the overexpression of TGF-β1 in the cartilage may be involved in the potential mechanism of the preventive and protective effects of RAL in osteoporotic OA. To the best of our knowledge, similar results have not been described in previous studies. We suggest that the potential mechanism of this effect may be attributed to the improvement of subchondral bone health by RAL. Alteration of subchondral bone was shown to play a key role in the progression of OA [43-45]. In the present study, micro-CT analysis indicated that the microstructural destruction of the subchondral bone was considerably aggravated in the model of osteoporotic OA in agreement with the results of previous studies [7,35,46]. The potential mechanism of this effect may be associated with accelerated bone resorption [43]. Col-I and TRAP are the markers of bone formation and osteoclast function, respectively [47]. In this study, the level of Col-I was decreased and the number of TRAP + osteoclasts was increased in the ACLT + OVX group compared with those in the OVX group, indicating that the combination of abnormal stress and estrogen deficiency further enhances the osteoclastic activity and accelerates bone loss thus leading to the aggravation of trabecular microstructural damage [43]. This effect was also reported in another surgically induced model of osteoporotic OA [35]. Conversely, RAL decreased the number of osteoclasts, increased bone mass, and alleviated the microstructural damage of trabecular, suggesting that RAL protects the health of the trabecular bone by modulating bone turnover, as described in previous studies [35,48]. As mentioned above, damage of the subchondral bone microstructure may enhance the expression of TGF-β1. Thus, RAL may indirectly reduce the level of TGF-β1 in the cartilage by improving the health of the subchondral bone. However, validation of this hypothesis requires additional molecular and biomechanical studies of the cartilage. Furthermore, RAL was shown to decrease the level of TGF-β1 by downregulating ALK1 in human fibroblasts in skin disease [49]. Nevertheless, it is not known whether RAL has a similar effect on the cartilage in osteoporotic OA.

This study has several limitations. We evaluated the levels of the target cytokines by immunohistochemistry and did not assess the mRNA levels of the target cytokines using real-time reverse transcription-polymerase chain reaction (RT-PCR) or evaluate protein levels with Western blotting analysis. Additionally, the influence of RAL on the modulation of the ALK/Smad pathways was not investigated. Hence, further molecular studies are necessary to determine the mechanism of the effect of RAL on the TGF-β1 signaling pathway in osteoporotic OA. Moreover, due to the differences in physiology and metabolism, the therapeutic effects of RAL in human OA need to be determined in clinical investigations.

## CONCLUSION

In conclusion, we have demonstrated that RAL significantly retards the cartilage degeneration, suppresses the overexpression of TGF-β1, and catabolic enzymes in the cartilage and improves the health of the subchondral bone in a model of osteoporotic OA in rats. The dose of RAL used in this study is equivalent to the clinical dose. Therefore, our findings provide support for the clinical application of RAL in the prevention and treatment in postmenopausal OA.

## Figures and Tables

**Supplementary table 1:**
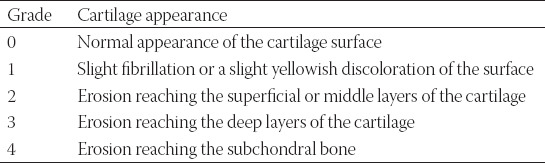
Macroscopic scoring system for cartilage lesion
